# Intraoral lipomas: A clinicopathological study of 43 cases, including four cases of spindle cell/pleomorphic subtype

**DOI:** 10.4317/medoral.22931

**Published:** 2019-05

**Authors:** Matheus-Ferreira Linares, Augusto-César-Leal-da Silva Leonel, Elaine-Judite-de Amorim Carvalho, Jurema-Freire-Lisboa de Castro, Oslei-Paes de Almeida, Danyel-Elias-da Cruz Perez

**Affiliations:** 1 School of Dentistry, Oral Pathology Unit, Universidade Federal de Pernambuco (UFPE), Recife, Pernambuco, Brazil; 2 Piracicaba Dental School, Oral Pathology, State University of Campinas (UNICAMP), Piracicaba, São Paulo, Brazil

## Abstract

**Background:**

The aim of this study was to describe the clinicopathological characteristics of 43 intraoral lipomas and classify them according to their microscopic variants.

**Material and Methods:**

All the cases of intraoral lipomas diagnosed at an Oral Pathology service were selected for the study. Clinical data, such as age, gender, location, time of evolution, clinical presentation, clinical hypothesis of diagnosis, and treatment, were collected from the clinical files.

**Results:**

Of the 43 cases analyzed, 24 (55.8%) occurred in women. The mean age was 77.4 years. The most affected site was the buccal mucosa (22 cases, 51.1%). The mean lesion size was 1.7 cm. Twenty-three cases (53.5%) were classified as simple lipoma, 14 (32.6%) as fibrolipoma, four (9.3%) as spindle cell/pleomorphic lipoma (SC/PL), one (2.3%) as lipoma of the salivary glands, and one (2.3%) as intramuscular lipoma. In one case of SC/PLs, lipoblasts were observed. No atypical lipoblasts or mitoses were noted. Lipoma was considered more often than other tumor histological subtypes among the clinical hypotheses of diagnosis when the final diagnosis was simple lipoma (*p*=0.01).

**Conclusions:**

Intraoral lipomas present different clinical presentation depending on the histological subtype. In SC/PLs, lipoblasts with vacuolated cytoplasm may be found and the presence of mature adipocytes is essential for diagnosis.

** Key words:**Lipoma, mouth, spindle cell lipoma, pleomorphic lipoma.

## Introduction

Lipomas are the most common benign mesenchymal neoplasms, occurring mainly in the dermis, especially the back, neck, armpit, and face. Their prevalence in the oral cavity is low, representing approximately 4.4% of all benign mesenchymal neoplasms of the oral cavity (1-3). Clinically, intraoral lipomas (OLs) usually appear as a painless, sessile or pedunculated nodule of soft consistency and long evolution time. Superficial lesions commonly present as yellowish nodules. The buccal mucosa is the most common site, followed by the tongue (2-5). Although rare, lipomas may occur in the maxilla or mandible, corresponding to 3% of all intraosseous lipomas of the body (6).

Microscopically, OLs are composed of mature adipocytes circumscribed by a thin fibrous capsule, which can be classified as simple lipoma, fibrolipoma, osteolipoma, intramuscular lipoma, angiolipoma, salivary gland lipoma, spindle cell/pleomorphic lipoma (SC/PL), chondrolipoma or myxoid lipoma (3,7-9). SC/PLs present identical cytogenetic features, representing different histological spectrum of a single disease (10).

Although OLs are not uncommon, there are few large series of cases published in the English-language literature. Thus, the objective of this study was to analyze the clinical and histopathological features of 43 cases of intraoral lipomas, including 4 cases of SC/PL.

## Material and Methods

This study is retrospective, observational, and descriptive, and it was approved by the Local Institutional Research Board (protocol# 44536715.8.0000.5208).

Between January 2000 and May 2017, all cases of lipoma diagnosed in the Oral Pathology Laboratory of the Universidade Federal de Pernambuco, Brazil, were selected for this study. Clinical data, such as patient age, gender, site, time of complaint, clinical presentation, clinical hypothesis of diagnosis, and treatment were recorded from the clinical charts. To confirm the diagnosis and classify the oral lipomas, all cases were microscopically reviewed on hematoxylin-eosin stained slides. Cases that were not located in the oral mucosa, did not present adequate tissue for revision, or did not represent a lipoma, were excluded from the study. According to microscopic features, lipomas were classified as simple lipoma, fibrolipoma, SC/PL, osteolipoma, intramuscular lipoma, angiolipoma, salivary gland lipoma, or chondrolipoma (1,3,5,8).

To confirm the diagnosis of SC/PL, immunohistochemical reactions were performed using 3-μm-thick histological sections on silanized slides using anti-S100 (polyclonal, dilution 1:10.000), vimentin (clone Vim 3B4, dilution 1:400), CD34 (clone QBEnd10, dilution 1:50), and specific-muscle actin (clone HHF35, dilution 1:800) antibodies. In these cases, to assess the cellular proliferation index, immunohistochemical reactions against Ki-67 (clone MIB1, Dako, dilution 1:100) were also performed. All antibodies were obtained from Dako (Glostrup, Denmark).

The data were analysed by descriptive statistics using IBM SPSS Statistics for Windows, version 20.0, with relative and absolute distributions of clinical and histopathological data. The clinical and histopathological variables were then analyzed using Fisher’s exact tests with a significance level of 5% (*p*<0.05).

## Results

During the study period, 55 cases out of 5,850 (0.94%) were lipomas. Twelve cases were excluded according to the exclusion criteria, two that were not located in the oral cavity and 10 without adequate tissue for histopathological review. Thus, 43 cases of intraoral lipomas were included in this study, corresponding to 0.73% of all lesions diagnosed in the Laboratory during the study period of 18 years. Of these, 24 (55.8%) occurred in women, while 19 (44.2%) affected men, with a female:male ratio of 1.2:1. The mean age was 77.4 years, ranging from 12 to 97, with 11 cases occurring in the eighth decade of life (*p*=0.005).

**Figure 1:**
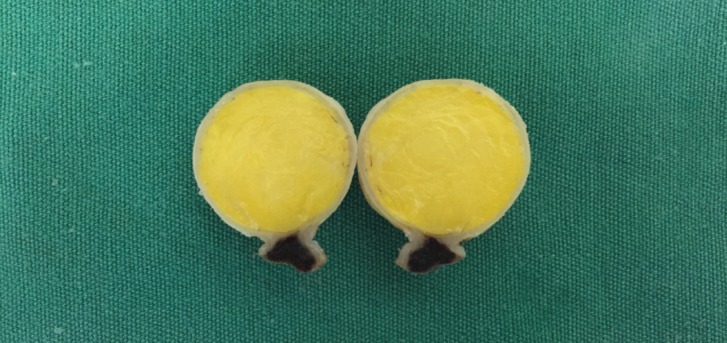
Macroscopic aspect of an oral lipoma of the buccal mucosa presenting as nodular and pedunculated lesion.

The most affected site was the buccal mucosa (22 cases, 51.1%), followed by the tongue (8 cases, 18.6%) and lower lip (7 cases, 16.3%) ( Table 1). The mean size of the lesions was 1.7 cm (range: 0.4–6.0 cm). All patients complained of a painless nodule, with a mean time of complaint reported by the patients of 38.7 months (range: 3–240 months). In 19 cases (44.2%), the lesion appeared as a nodule covered by normal-colored mucosa and in seven (16.3%) the nodule was yellowish. In 17 cases (39.5%) this information was not available. Most cases (27, 62.8%) appeared as superficial lesions and 11 (25.6%) as submucosal nodules. This information was missing in 5 cases (11.6%). Fifteen cases (34.9%) were sessile nodules, while 11 (25.6%) appeared as pedunculated lesions, and in 17 cases (39.5%), this data was unavailable. Information about the clinical hypothesis of diagnosis was available in 35 cases, 20 (57.1%) included lipoma as one of the diagnostic hypotheses and lipoma was not considered in 15 cases (42.9%). Fibrous hyperplasia was the second most common lesion included among the diagnostic hypotheses (12 cases, 34.3%). All cases underwent surgical excision (Fig. 1).

**Table 1:**
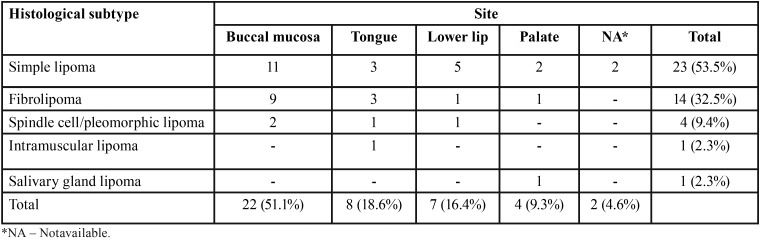
Distribution of the histological subtypes of oral lipomas according to anatomical site.

**Figure 2:**
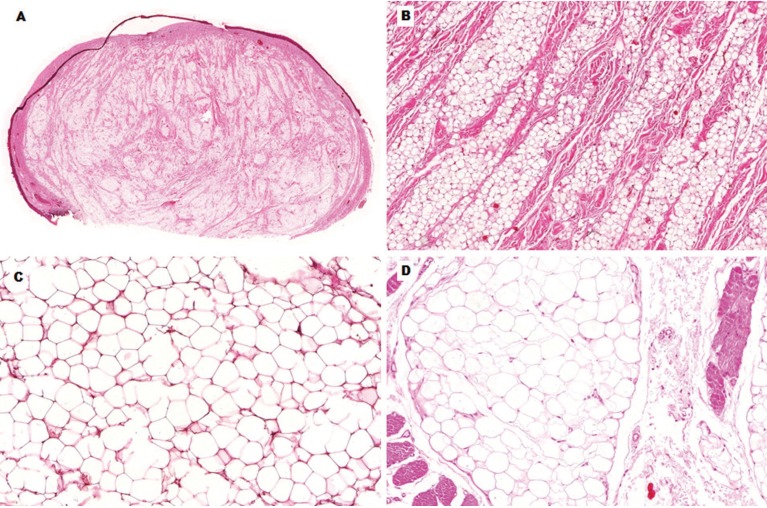
Microscopic features of histological subtypes of lipomas of the mouth. A, Panoramic view of a well-circumscribed nodular lesion, located adjacent to the oral epithelium (HE, 10x). B, Mature adipocytes interspersed by dense and thick bundles of fibrous connective tissue, characterizing a fibrolipoma (HE, 100x). C, Simple lipoma presenting well- differentiated adipocytes (HE, 200x). D, Intramuscular lipoma showing well-differentiated adipocytes interspersed by skeletal muscle fibers (HE, 200x).

**Figure 3:**
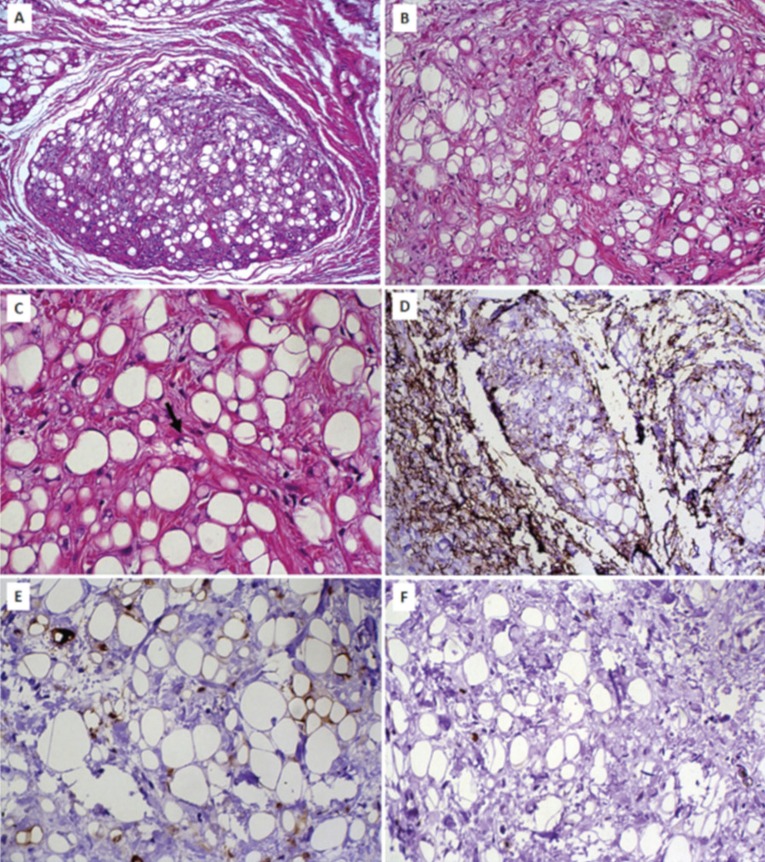
Microscopic and immunohistochemical features of spindle cell/pleomorphic lipoma. A, Mature adipocytes in varying sizes interspersed by bland spindle cells arranged in a loose stroma (HE, 100x). B, High-power view of the features observed in A (HE, 200x). C, Presence of lipoblasts, one of them vacuolated (arrow), well-differentiated adipocytes, and spindle cells arranged in a fibrous connective tissue (HE, 400x). D, Spindle cells positive for CD34 (streptavidin-biotin-peroxidase method, 200x). E, Spindle cells negative for S-100 protein. Note positivity only in mature adipocytes (streptavidin-biotin-peroxidase method, 400x). F, Less than 1% of Ki-67-positive tumor cells (streptavidin-biotin-peroxidase method, 200x).

Histologically, 23 cases (53.5%) were classified as simple lipoma, 14 (32.6%) as fibrolipoma, four (9.3%) as SC/PL, one (2.3%) as salivary gland lipoma, and one (2.3%) as intramuscular lipoma (Fig. 2). The SC/PLs presented spindle-shaped cells frequently arranged in a loose and myxoid stroma interspersed by mature adipocytes of varying sizes. Immunohistochemical analysis of these lipomas revealed that the spindle cells were positive for vimentin and CD34, and negative for S-100 protein and muscle-specific actin (Fig. 3). Mature adipocytes were positive for S-100, and less than 1% of the tumor cells were positive for Ki-67 (Fig. 3). In one case of SC/PLs, lipoblasts were observed, with some of them being vacuolated (Fig. 3). No atypical lipoblasts, mitoses, or necrosis were found.

In all cases that appeared as a yellowish nodule, lipoma was considered in the differential diagnosis. However, lipoma was not considered among the clinical hypotheses of diagnosis in 57.1% of the cases that showed normal-colored mucosa (*p*=0.04). Lipoma was considered more often among the clinical hypotheses of diagnosis when the final diagnosis was simple lipoma compared to the other tumor histological subtypes (*p*=0.01). Likewise, only cases with a final diagnosis of simple lipoma appeared as yellowish nodules (*p*=0.01).

## Discussion

Although lipomas are common in other regions of the body, OLs represent only 0.27%–1.7% of all oral lesions (4,11), similar to the prevalence observed in the present study. The gender predilection is variable, but a higher prevalence in men, with male:female ratios ranging from 1.19:1 to 2.75:1, was reported (12,13). The higher male:female ratio (2.75:1) was observed in a study performed in the records from the Armed Forces Institute of Pathology in USA (13), which could explain the high prevalence in men. In other studies, however, there was a predilection for women, with female:male ratios ranging from 1.18:1 to 7:1 (3,14). In the present study, there was a slight predilection for women, with a female:male ratio of 1.2:1.

OLs occur more frequently in patients between the sixth and seventh decades of life (2,4,9,11). Although rare, they may also affect pediatric patients (2,5,9,13). At diagnosis, the mean age of patients varies from 50.2 to 59.7 years (5,11). In the present series, only one case occurred in a pediatric patient, and lipomas were significantly more common in elderly patients, with a mean age of 77.4 years.

The most common sites of OLs are the buccal mucosa, tongue, and lips (2,4,5,9,11), as observed in the present series. OLs usually appear with a mean size of 2 cm (1,3,5,7,9,13), comparable to that in the present study. However, the size can range from very small lesions, measuring 0.2 cm, to large tumors, measuring approximately 10 cm (4,7). OLs have a slow growth, with a patient-reported evolution time of complaint ranging from 4 days to 37 years (3,9). In this study, the mean duration between the patient perception and the search for a diagnosis was 38.7 months.

Clinically, OLs commonly appear as submucosal or superficial nodules with an intact surface and coloration ranging from yellowish to normal mucosa. Oral epidermoid cyst, oral lymphoepithelial cyst, salivary gland tumors, and other benign mesenchymal neoplasms should be included in the differential diagnosis of OLs (1,11). The present series revealed that the color of the tumor at the clinical presentation is fundamental in the inclusion of lipomas for the differential diagnosis of submucosal or superficial intraoral nodules. In addition, the histological subtype significantly affects the clinical presentation of the lesion, as shown in this study. Only cases classified as simple lipoma appeared as yellowish nodules.

The most common histopathologic subtypes are simple lipomas and fibrolipomas (1,2,4,5,7,9). Some studies have reported similar lipoma/fibrolipoma ratios (1,5,11,13), while others reported simple lipoma to be more common (2,3,4,11,13), as observed in the present study. In this series, simple lipoma and fibrolipoma corresponded to 85.9% of the cases. Although most lipomas are simple to diagnose, some histological subtypes may represent a challenge, especially SC/PLs. SC/PLs are rare in the oral cavity, with variable prevalence among studies, representing up to 9.8% of all OLs (15), as observed in this series. These histopathologic subtypes present similar biological behavior and prognosis (9).

Oral SC/PLs show a distinctive clinical profile compared to the other subtypes of lipomas, with most cases occurring in the tongue (15). In this series, two cases affected the buccal mucosa and one case each occurred on the tongue and lip. Microscopically, fat-forming solitary fibrous tumor, cellular angiofibroma, mammary-type myofibroblastoma, neurofibroma, myxofibrosarcoma, atypical spindle cell lipomatous tumor, and atypical lipomatous tumor/well-differentiated liposarcoma may be considered in the differential diagnosis of SC/PL (16,17). The presence of lipoblasts, some of them vacuolated, as observed in the present study, may lead to an incorrect diagnosis of liposarcoma, especially in atypical lipomatous tumor/well-differentiated liposarcoma. The occurrence of adipocytes in variable amounts is an essential finding for diagnosis. The ratio of mature adipocytes to spindle cells can vary significantly. In rare cases, small amounts of mature adipocytes are present in SC/PLs, which can represent an additional diagnostic challenge (18). It is important to note that lipoblasts may be found in benign lipogenic tumors, including SC/PL. However, the presence of atypical lipoblasts and mitoses do not support the diagnosis of SC/PL (19). None of these features were found in the current cases. Atypical spindle cell lipomatous tumor (ASCLT) represents a distinct group of adipocytic neoplasms that present an intermediate biological potential, located most commonly in limbs (10,20). Microscopically, ASCLT is composed of relatively uniform spindle cells interspersed by a variable number of mature-appearing adipocytes in varying sizes. The spindle cells show varying degrees of nuclear atypia across different tumors, with about 30% of the cases presenting prominent and diffused atypia (10). In contrast to SC/PL, ASCLT often presents ill-defined margins and infiltration of adjacent tissue in about 30% of the cases. Additionally, the presence of ropey collagen fibers, common in SC/PL and present in the current cases, is rare in ASCLT (21,22).

In the present cases of SC/PL, the tumor spindle cells were positive for CD34 and negative for S-100 protein. Despite this finding, it is important to highlight that CD34 is expressed in several other soft tissue tumors, including non-lipomatous and lipomatous tumors, such as fat-forming solitary fibrous tumor and ASCLT. The presence of hemangiopericytomatous pattern, simulating a fat-forming solitary fibrous tumor, is rarely observed in SC/PL. Moreover, ropey collagen is not common in solitary fibrous tumor, and STAT6 is negative in SC/PL (20). Liposarcomas usually present higher Ki-67 index than benign adipocytic tumors (23). However, a low Ki-67 index did not exclude a malignant adipocytic tumor, mainly well-differentiated malignant neoplasms. Thus, although the immunohistochemical profile is useful, the diagnosis of SC/PL should be based mainly on histological features (15). Molecular and cytogenetic features are also not definitive for diagnosis of SC/PL. Loss of pRb (deletion of 13q14), observed in SC/PL, also occurs in about 50% of the ASCLT. In addition, no amplification of MDM2 and CDK4 is observed in both SC/PL and ASCLT (10).

The treatment of OLs consists in simple surgical excision, with excellent prognosis, and no recurrences (1,3,4,9,11). In this series of 43 cases, OLs presented a slight predilection for women, more commonly in elderly patients. OLs usually appear as a painless nodule; in most cases covered by a normal-colored mucosa. In fact, regardless of the classical yellowish color, lipomas should be considered in the differential diagnosis of superficial or submucosal oral nodules. Particularly in SC/PLs, lipoblasts with vacuolated cytoplasm may be found and the presence of mature adipocytes is essential for diagnosis.
